# The Potential Role of an Adjunctive Real-Time Locating System in Preventing Secondary Transmission of SARS-CoV-2 in a Hospital Environment: Retrospective Case-Control Study

**DOI:** 10.2196/41395

**Published:** 2022-10-18

**Authors:** Min Hyung Kim, Un Hyoung Ryu, Seok-Jae Heo, Yong Chan Kim, Yoon Soo Park

**Affiliations:** 1 Division of Infectious Diseases, Department of Internal Medicine Yongin Severance Hospital Yonsei University College of Medicine Yongin-si Republic of Korea; 2 Division of Planning and Management, Office of Medical Information Technology Yongin Severance Hospital Yonsei University College of Medicine Yongin-si Republic of Korea; 3 Division of Biostatistics Department of Biomedical Systems Informatics Yonsei University College of Medicine Seoul Republic of Korea

**Keywords:** real-time locating system, COVID-19, contact tracing, secondary transmission, SARS-CoV-2

## Abstract

**Background:**

There has been an increasing demand for new technologies regarding infection control in hospital settings to reduce the burden of contact tracing.

**Objective:**

This study aimed to compare the validity of a real-time locating system (RTLS) with that of the conventional contact tracing method for identifying high-risk contact cases associated with the secondary transmission of SARS-CoV-2.

**Methods:**

A retrospective case-control study involving in-hospital contact cases of confirmed COVID-19 patients, who were diagnosed from January 23 to March 25, 2022, was conducted at a university hospital in South Korea. Contact cases were identified using either the conventional method or the RTLS. The primary endpoint of this study was secondary transmission of SARS-CoV-2 among contact cases. Univariate and multivariable logistic regression analysis comparing test positive and versus negative contact cases were performed.

**Results:**

Overall, 509 and 653 cases were confirmed by the conventional method and the RTLS, respectively. Only 74 contact cases were identified by both methods, which could be attributed to the limitations of each method. Sensitivity was higher for the RTLS tracing method (653/1088, 60.0%) than the conventional tracing method (509/1088, 46.8%) considering all contact cases identified by both methods. The secondary transmission rate in the RTLS model was 8.1%, while that in the conventional model was 5.3%. The multivariable logistic regression model revealed that the RTLS was more capable of detecting secondary transmission than the conventional method (adjusted odds ratio 6.15, 95% CI 1.92-28.69; *P*=.007).

**Conclusions:**

This study showed that the RTLS is beneficial when used as an adjunctive approach to the conventional method for contact tracing associated with secondary transmission. However, the RTLS cannot completely replace traditional contact tracing.

## Introduction

Human history is characterized by the incessant influence of infectious diseases, with viruses being the most successful contender [1]. Viral diseases dominate on the World Health Organization’s list of top priorities of concern [2]. SARS-CoV-2 is one of the novel viruses exerting unprecedented influence on the world’s population due in part to a lack of knowledge. In the early stage of the COVID-19 pandemic, nonpharmaceutical approaches, such as mask wearing and isolating infected patients, comprised the predominant methods of preventing the disease from spreading [3,4]. Despite the development of pharmaceutical agents, such as vaccines and therapeutic antiviral drugs, these nonpharmaceutical measures are not obsolete because of the emergence of new variants and the waning effect of vaccines [5-8]. The importance of nonpharmaceutical measures is emphasized in the hospital environment where immunocompromised populations, such as patients with cancer and older patients, are concentrated.

Contact tracing is an important strategy to keep disease transmission under control by isolating high-risk contacts who eventually present with the disease. Contact tracing is a time- and labor-consuming procedure, the efficiency of which is dependent on the commitment of the infection control personnel and the presence of beneficial adjunctive tools. Moreover, the importance of this method could be diminished in the era of “living with COVID-19,” wherein efforts to confine the spread of the disease are diluted with the weakening of disease severity. However, in a hospital setting, simplifying rather than eliminating the contact tracing effort is required. SARS-CoV-2 is a complicated virus to deal with, especially in the hospital setting, without sufficient data regarding its mode of transmission [9-13]. Furthermore, a variety of common transmissible diseases that require contact tracing can spread in hospitals. For most of those diseases, the distance and duration of exposure are of primary importance when deciding the high-risk contacts of a confirmed patient [14-17].

Technological efforts, such as a real-time locating system (RTLS), could be an option to overcome the limitation set by the conventional method. One type of RTLS involves radio-frequency identification (RFID) and a Wi-Fi tracking system. RFID calculates the distance and duration of human-to-human interaction by analyzing the signals from RFID tags worn by users, which are captured by exciters installed in hospital wards and working places [18]. Using this technology, the quantity of interaction affordable, regardless of the number of contacts, can be determined [19]. Evidence of the validity of this technology in a hospital setting is accumulating, despite its privacy concerns and cost-benefit issues [20,21]. The efficiency of this technology for preventing the spread of transmissible diseases needs to be elucidated.

The aim of this study was to evaluate the validity of the RTLS compared with the conventional contact tracing method to identify high-risk contacts associated with the secondary transmission of SARS-CoV-2. Furthermore, we attempted to characterize the factors associated with secondary SARS-CoV-2 transmission using both methods.

## Methods

### Setting

This study was conducted at a Yongin Severance University–affiliated hospital in South Korea, with 580 beds and an 82% average occupancy rate annually. This institution had RTLS location sensors since its opening in 2020. All health care workers and inpatients were issued RTLS tags that detected their locations.

From the time COVID-19 was declared a pandemic, health care workers, hospitalized patients, and caregivers in this hospital were monitored for the presence of symptoms suggestive of COVID-19 on a daily basis. Caregivers included the patients’ family members or privately employed carers. Employees were mandated to report COVID-19–related symptoms through a mobile app at least once a day. Hospitalized patients and caregivers were obligated to take screening reverse transcription polymerase chain reaction (RT-PCR) tests ahead of admission, and COVID-19–related symptoms were closely monitored by attending nurses, which were recorded electronically. SARS-CoV-2 polymerase chain reaction (PCR) tests were conducted for those who developed COVID-19–related symptoms, and quarantine measures were implemented for individuals who tested positive for SARS-CoV-2. Subsequent contact tracing was carried out by the infection control office staff and the digital information team, with stratification of contacts according to the level of exposure. All contacts regardless of the exposure level were recommended to get tested for SARS-CoV-2 at least once, with specific emphasis on high-risk contacts within 14 days after exposure or on the development of symptoms. Postexposure measures, such as quarantine, were implemented for those who were identified as contacts at the discretion of staff in the infection control office. As RTLS data were collected only for research purposes, no postexposure interventions were implemented based solely on RTLS data.

### Study Design and Identification of Contact Cases

A retrospective case-control study involving in-hospital contact cases of confirmed COVID-19 patients, who were diagnosed from January 23 to March 25, 2022, was conducted. All contact cases of health care workers, and inpatients and their caregivers, identified either by the conventional method or the RTLS, were included in this study. The participants were followed up from the date of contact to 14 days following the last contact or the date of a follow-up SARS-CoV-2 PCR test.

Contact tracing started 2 days prior to the symptom onset or positive PCR test result of a COVID-19–confirmed patient. The conventional method of contact tracing starts with an in-person interview, followed by reviewing electronic medical records and monitoring closed circuit surveillance camera feeds based on the information acquired in the interview. RTLS-based contact tracing was performed separately by the digital information team. When a patient tested positive, the digital information team extracted data from the RTLS to identify close contact cases. The radio-frequency RTLS sensors that can detect signals within a radius of 20 meters were located in every room in the hospital and at every 10 meters in open spaces. Hospital staff and inpatients were required to wear RTLS tags at all times. Signals were emitted from the tags every 1 to 3 seconds to confirm the presence of individuals in a room or confirm the distance between individuals through tag-tag signal interaction. When 2 individuals got close enough to a designated distance, the calculation of contact time was started to obtain the cumulative contact time between the 2 individuals. Generally, it took less than 30 minutes to draw data from the RTLS.

The level of exposure was determined based on a Centers for Disease Control and Prevention (CDC) guideline [22]. The CDC provides information on the transmission risk of COVID-19 among contacts according to the level of exposure, and recommends actions to prevent disease transmission. High-risk exposure was defined as close contact with confirmed patients within 2 meters for more than 15 minutes without adequate mask wearing, or physical contact without wearing gloves or protective gowns. Intermediate-risk exposure was defined as contact with confirmed patients within 2 meters for more than 15 minutes with moderate protective equipment. Low-risk exposure was defined as contact with confirmed patients with adequate protective gear, or contact outside the range of high-risk exposure without protective equipment.

The primary endpoint of this study was secondary transmission of SARS-CoV-2 among contact cases. Secondary transmission was assumed when there was a positive conversion of the SARS-CoV-2 PCR test following a negative test result within 14 days of contact. Those without previous test results were included as well, unless they had evidence of other sources of infection, such as being simultaneously diagnosed with index patients, having a known familial transmission, or showing COVID-19–related symptoms.

### Exclusion Criteria

The exclusion criteria were as follows: (1) no identifiable age or gender information; (2) no follow-up PCR results; and (3) distance of more than 3 meters from index patients among RTLS-confirmed cases.

### Data Collection

The data of in-hospital–confirmed COVID-19 patients were collected retrospectively. Data, including age, gender, vaccination history (including number of vaccinations and days passed from the last vaccination), follow-up SARS-CoV-2 PCR test results, date of diagnosis in case of a positive result, closest exposure distance, duration of exposure within a distance of 2 meters, whether personal protective equipment was used, mask-wearing habit, type of occupation, type of occupation of index patients, date of the last contact, whether the room was shared with index patients, methods used to identify contact cases and postexposure measures, and whether tags were worn, were collected by reviewing the records acquired for contact tracing. We filled up some part of the data regarding wearing masks through estimation based on the hospital policy, when fact-checking was impossible due to long hours of exposure.

### SARS-CoV-2 RT-PCR

SARS-CoV-2 RT-PCR was performed with nasopharyngeal swab samples collected from participants. The MagNA Pure 96 System (Roche Diagnostics) was used to extract RNA from nasopharyngeal swabs in viral transport media, according to the manufacturer’s instructions. The extracted RNA was then subjected to the Allplex SARS-CoV-2 Assay, which targets 4 genes in a single tube (E, N, RdRp, and S genes) to detect SARS-CoV-2 infection, according to the manufacturer’s instructions. PCR amplification was performed using the CFX96 real-time PCR detection system (Bio-Rad Laboratories).

### Statistical Methods

Analyses comparing secondary transmission cases and test-negative cases were performed. We allowed the inclusion of multiple episodes of the same individuals because the nature of contact cases was different each time. Baseline characteristics were compared using the Mann-Whitney *U* test, independent samples *t* test, or ANOVA for continuous variables, and the χ^2^ test or Fisher exact test for categorical variables. Continuous variables were expressed as means or medians (IQRs) and categorical variables as numbers with percentages for the description of baseline characteristics. Logistic regression was used to identify factors related with secondary transmission adjusting for relevant variables with a *P* value <.05 in univariate analysis. Cumulative hazard curves were created using the Kaplan-Meier method, and the hazards of detecting secondary transmission for each model were compared according to the study date using the log-rank test. Sensitivity analysis was conducted with participants having follow-up PCR results within 14 days available to identify either consistency of or differences in the magnitude of the effect. Missing values were removed from the analysis. All statistical analyses were performed using SPSS software version 26 (IBM Corp). Two-sided *P* values <.05 were considered statistically significant.

### Ethics Statement

This study was approved by the Institutional Review Board of Yonsei University Health System Clinical Trial Centre, and the study protocol adhered to the tenets of the Declaration of Helsinki. As the study was retrospective, the Institutional Review Board waived the requirement for written informed consent from the participants (approval number: 9-2022-0027; approved on April 22, 2022).

## Results

### Study Participants

**Figure 1:**
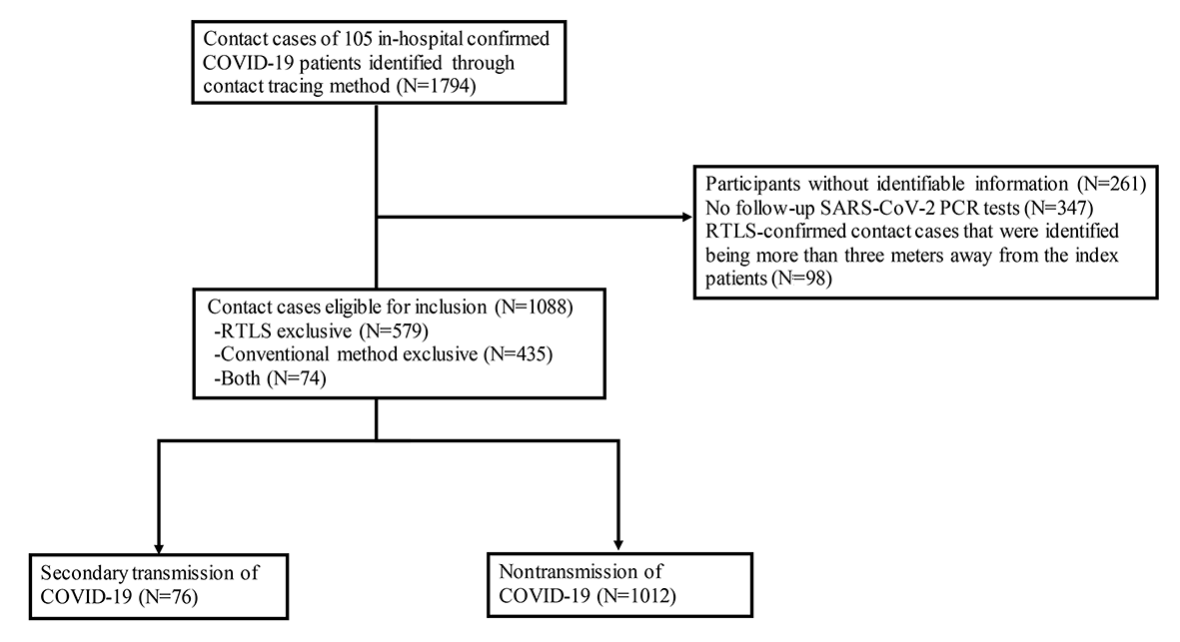
Study flow of enrollment. PCR: polymerase chain reaction; RTLS: real-time locating system.

Among 1794 cases identified by the methods described above, 261 cases without age or gender information, 98 RTLS-confirmed cases that were identified more than 3 meters away from index patients, and 347 cases without follow-up test results were excluded. As a result, 1088 contact cases were included in the analysis. Among 79 cases that tested positive for SARS-CoV-2 within 14 days of exposure, 3 cases were excluded from secondary transmission owing to the presence of other sources of transmission (Figure 1).

The baseline characteristics of all participants are presented in the following text. The mean age of the participants was 41.5 (SD 17.5) years, with 25.3% (275/1088) being male participants. Among contacts, 70.7% (769/1088) were health care workers, and among these, 6.2% (48/769) were doctors, 71.5% (550/769) were nurses, and 22.2% (171/769) were others. The percentage of those vaccinated at least once prior to contact was 83.8% (741/884), with a median of 82 (IQR 54-82) days from the last vaccination. The median contact duration was 240 (IQR 41-1675.8) minutes. Among those who were designated as index patients, 80.3% (843/1051) were health care workers. Furthermore, room sharing was confirmed in 71.9% (736/1023) of cases in the hospital. There were 63.5% (436/686) high-risk contact cases, 28.9% (198/686) intermediate-risk contact cases, and 7.6% (52/686) low-risk contact cases (Multimedia Appendix 1).

### Contact Cases Identified by the RTLS or the Conventional Method

Among the 1088 cases involving 764 participants, 76 cases involving 65 participants resulted in secondary transmission, while 1012 cases involving 730 participants remained negative for SARS-CoV-2 (Figure 1). Only 74 contact cases were identified by both methods, while 509 and 653 cases were confirmed by the conventional and RTLS methods, respectively. The factors associated with RTLS detection against the conventional method were identified. Younger age (37.6, SD 14.5 vs 47.6, SD 19.6 years; *P*<.001), being a health care worker (88.5% vs 60.3%; *P*<.001), being a health care worker who was an index patient (91.4% vs 62.4%; *P*<.001), room sharing (75.5% vs 69.9%; *P*=.04), mask wearing (26.5% vs 41.6%; *P*<.001), and being exposed for long durations (391 [IQR 64-1804] vs 33 [IQR 10-240] minutes; *P*<.001) were associated with RTLS detection. The level of exposure should be interpreted with caution, considering the large number of missing values (Table 1). The absence of records on exposure time largely contributed to the missing values of exposure levels in the conventional method. Analysis involving secondary transmission cases alone revealed that more health care workers were detected by the RTLS exclusive method (Multimedia Appendix 2).

**Table 1:** Baseline characteristics of participants based on the contact tracing method.

Characteristic				RTLS^a^ method^b^ (n=653)		Conventional method^b^ (n=509)		*P* value
Age (years), mean (SD)				37.6 (14.5)		47.6 (19.6)		<.001
Sex (male), n (%)				164 (25.1)		134 (26.3)		.64
**Exposure duration (minutes), median (IQR)**				391 (64-1804)		33 (10-240)		<.001
	Unknown^c^, n		2		344			
**Personal protective equipment used**								
	Mask, n (%)		158 (26.5)		207 (41.6)		<.001	
	Gloves, n (%)		0 (0.0)		6 (3.6)		.59	
	Face shield, n (%)		0 (0.0)		1 (0.6)		>.99	
	Unknown^c^, n		56		11			
**Mask-wearing consistency^d^**								
	At all times, n (%)		23 (92.0)		138 (84.7)		.33	
	More than 50%, n (%)		1 (4.0)		10 (6.1)		>.99	
	Less than 50%, n (%)		1 (4.0)		15 (9.2)		.70	
	Unknown^c^, n		628		346			
**Level of exposure**								
	High, n (%)		422 (70.9)		54 (33.3)		<.001	
	Intermediate, n (%)		161 (27.1)		61 (37.7)		<.001	
	Low, n (%)		12 (2.0)		47 (29.0)		<.001	
	Unknown^c^, n		58		347			
**Type of occupation**								<.001
	**Health care worker, n (%)**		525 (88.5)		284 (60.3)			
		Doctor, n (%)	26 (4.4)		23 (4.9)			
		Nurse, n (%)	388 (65.4)		188 (39.9)			
	**Patient, n (%)**		68 (11.5)		187 (39.7)			
		Patient, n (%)	68 (11.5)		130 (27.6)			
		Caregiver, n (%)	0 (0.0)		57 (12.1)			
	Unknown^c^, n		60		38			
**Type of occupation of the index patient**								<0.001
	Health care worker, n (%)		597 (91.4)		294(62.4)			
	Patient, n (%)		56 (8.6)		177 (37.6)			
	Unknown^c^, n		0		38			
**Vaccination status**								
	Vaccinated more than once, n (%)		493 (81.9)		294 (86.5)		.07	
	Days from last vaccination^e^ (days), median (IQR)		83 (68-196)		60 (38-170)		<.001	
	Unknown^c^, n		51		169			
**Postexposure measure**								
	Quarantined, n (%)		23 (69.7)		127 (65.8)		.06	
	Monitored actively, n (%)		8 (24.2)		34 (17.6)		.31	
	Monitored passively, n (%)		2 (6.1)		32 (16.6)		.12	
	Unknown^c^, n		620		346			
Secondary transmission, n (%)				53 (8.1)		31 (6.1)		.06
**Room sharing, n (%)**				450 (75.5)		348 (69.9)		.04
	Unknown^c^, n		57		11			
Compliance with tag wearing, n (%)				653 (100.0)		246 (48.3)		<.001

^a^RTLS: real-time locating system.

^b^Cases included in both the RTLS and conventional methods were handled as duplicate values.

^c^Unknown represents the number of missing values.

^d^Extent to which each participant conforms to the mask-wearing precaution.

^e^ Days passed from the last vaccination.

### Secondary Transmission Among Contact Cases Identified by the RTLS or the Conventional Method

**Figure 2:**
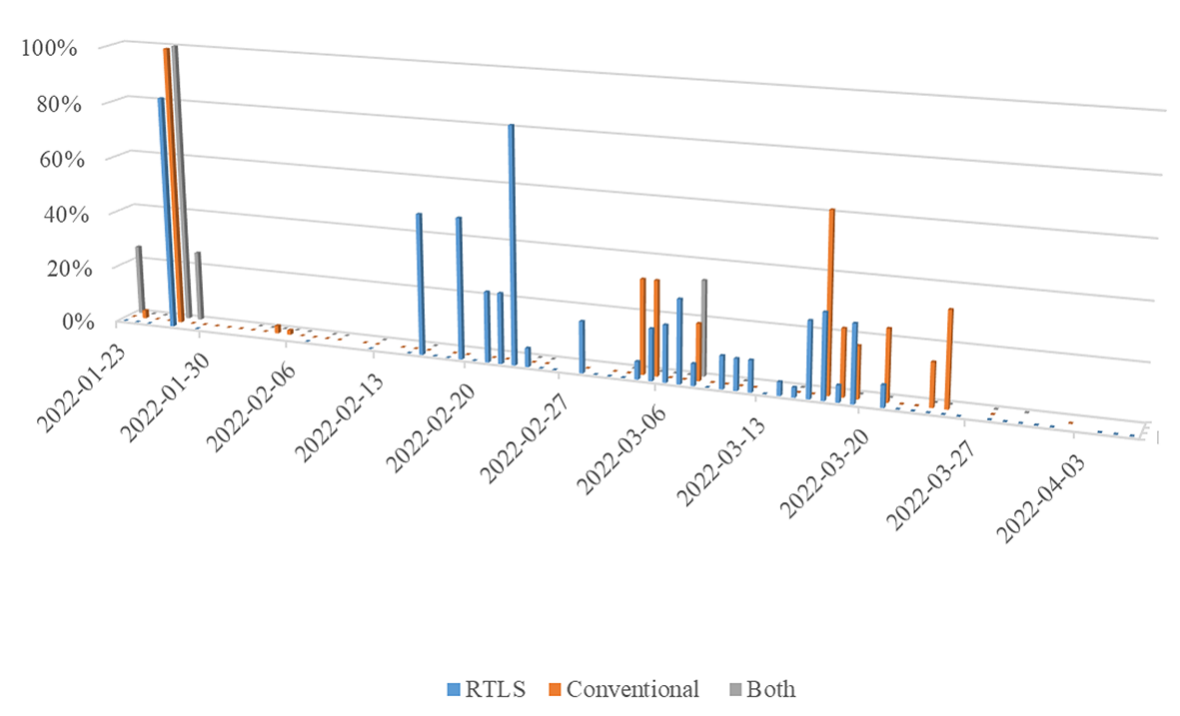
Secondary transmission rate calculated against contacts identified by each method according to the date of diagnosis. The secondary transmission rate was defined as cases of secondary transmission against contacts according to the date of index patients' confirmation. The average secondary transmission rate calculated against contacts identified exclusively by the RTLS was 10.6%, while that calculated against contacts identified exclusively by the conventional method was 7%. "Both" denotes cases identified by both the RTLS and conventional method. RTLS: real-time locating system.

The baseline characteristics of secondary transmission cases are described in Multimedia Appendix 1. Overall, the secondary transmission rate was 7.0% when all contact tracing methods were combined. The secondary transmission rate in the RTLS model was 8.1%, while that in the conventional method model was 5.3% (Table 2). The results spread out according to the confirmed date are presented in Figure 2, which shows a higher contribution of the RTLS than the conventional method in detecting secondary transmission.

We calculated the odds ratio (OR) for the secondary transmission group, with the group that tested negative for SARS-CoV-2 as a control. Variables with clinical significance and statistical significance in the univariate analysis were included in the multivariate analysis. The adjusted odds ratios (aORs) for clinically relevant variables and for variables with statistical significance in the univariate analysis revealed that male gender (aOR 0.11, 95% CI 0.01-0.53; *P*=.03), longer duration from the last vaccination (aOR 1.04, 95% CI 1.01-1.07; *P*=.006), and using the RTLS as the contact tracing method (aOR 6.15, 95% CI 1.92-28.69; *P*=.007) were associated with secondary transmission (Table 3). The Kaplan-Meier curve showed increased detection of secondary transmission among contact cases identified by the RTLS toward the end of the study period (Multimedia Appendix 3). Moreover, a subgroup analysis involving contact cases with available follow-up PCR tests within 14 days produced similar results (Multimedia Appendix 4).

The difference in cumulative contact duration was not statistically significant between the groups. The median contact duration was 630 [IQR 72.5-1510.5] minutes for the cases having secondary transmission versus 240 [IQR 41-1678] minutes for the controls (Multimedia Appendix 1). There were 3 cases of secondary transmission with 15 minutes of contact within 2 meters. All 3 were identified by the RTLS, and the time was precisely calculated. None of the cases were involved in aerosol-producing procedures.

**Table 2:** Comparison of the performance of each contact tracing method and the methods combined.

Variable	RTLS^a^ method			Conventional method			Both methods^b^	
	Detected	Not detected	Detected		Not detected	Detected		Not detected
Identified contact cases (N=1088), n (%)	653 (60.0)	435 (40.0)	509 (46.8)		579 (53.2)	74 (6.8)		1,014 (93.2)
Secondary transmission (N=76), n (%)	53 (69.7)	23 (30.3)	27 (35.5)		49 (64.5)	4 (5.3)		72 (94.7)
Secondary transmission rate^c^, %	8.1	N/A^d^	5.3		N/A	5.4		N/A

^a^RTLS: real-time locating system.

^b^“Both methods” denotes cases identified by both the RTLS and conventional methods.

^c^Secondary transmission rate was defined as cases of secondary transmission against contact cases identified by each method.

^d^N/A: not applicable.

**Table 3:** Logistic regression analysis for identifying the risk factors for SARS-CoV-2 secondary transmission.

Variable	Univariate analysis			Multivariable analysis^a^			
	OR^b^	95% CI	*P* value	OR^c^	95% CI		*P* value
Age	0.97	0.93-1.00	.12	1.00		0.96-1.05	.90
Male (reference: female)	0.11	0.01-0.50	.03	0.11		0.01-0.53	.03
Days from the last vaccination^d^ (days)	1.05	1.02-1.07	.001	1.04		1.01-1.07	.006
Room sharing	1.96	0.85-5.32	.14	2.72		0.40-14.42	.26
Mask wearing	0.55	0.23-1.19	.15	2.20		0.35-9.86	.34
RTLS^e^ (reference: conventional)	5.94	2.09-24.92	.004	6.15		1.92-28.69	.007

^a^Logistic regression was used to calculate the risk of secondary transmission. Variables with clinical significance and statistical significance in the univariate analysis were included in the model.

^b^OR: odds ratio.

^c^Adjustment for all the variables involved in the univariate model.

^d^Days passed from the last vaccination.

^e^RTLS: real-time locating system.

## Discussion

### Principal Findings

This study suggested that the RTLS has an added benefit for identifying close contact cases associated with secondary transmission of SARS-CoV-2 by identifying 64.5% (49/76) additional cases that were not detected by the conventional method. The RTLS had a higher power than the conventional method for detecting high-risk contact cases that subsequently developed COVID-19. However, the technology may not be used separately from the conventional method owing to moderate sensitivity.

### Comparison With Prior Work

The utility of the RTLS for tracing contacts of multiple transmissible diseases in a hospital setting has been explored in previous studies. Researchers suggested that the RTLS has moderate to high sensitivity but a low positive predictive value when compared with the conventional tracing method in detecting contact cases for droplet-transmitted diseases such as COVID-19 [21,23]. Our study showed that sensitivity was higher for the RTLS tracing method (653/1088, 60.0%) than the conventional tracing method (509/1088, 46.8%) considering all contact cases identified by both methods. The value is not acceptably high for its use as a single method for contact tracing. However, this method showed promising results in terms of efficiency. To the best of our knowledge, this study is the first to discuss the efficiency of the RTLS for detecting high-risk contact cases associated with secondary SARS-CoV-2 infection. When all methods were combined, the secondary transmission rate among health care staff and patients was 7.0%, while that identified by the RTLS was 8.1% and that identified by the conventional method was 5.3%. The average secondary transmission rate was lower than that in the community setting [24-26]. Based on the fact that the denominator involves contact cases identified by methods with no known gold standard, a higher secondary transmission rate may mean higher efficiency of the contact tracing method. The logistic regression model showed that the odds of detecting secondary transmission cases was higher for the RTLS than the conventional method. This may indicate that the RTLS is not inferior to conventional methods in performing contact tracing, especially when considering its time-saving characteristics.

The sensitivity of the RTLS was lower when the conventional method was used as a reference (74/653, 11.3%). This discrepancy is associated with inherent limitations of the RTLS or conventional method. The efficacy of the RTLS is dependent on the commitment of participants to wearing tags and the frequency of the signal exciter [27]. As was shown in this study, the tag-wearing behavior and location of participants were associated with the discrepancy. Working as a young nurse was associated with RTLS detection owing to a favorable tag-wearing behavior. Conventional contact tracing relies heavily on a person’s memory, which might be subjective and inaccurate. It tends to be biased toward identifying vulnerable contacts, such as hospitalized patients, which may be another explanation for the discrepancy. The RTLS would be beneficial when used for highly transmissible infectious diseases because of its time-saving property, which can help detect more high-risk contacts associated with secondary transmission. The Kaplan-Meier curve showed a trend of increased detection of secondary transmission cases through the RTLS toward the end of our research when an increasing proportion of Omicron variant cases was being reported on a weekly basis. However, our results suggest that the RTLS cannot be used alone for tracing contacts. Although the efficacy of the RTLS as an adjunctive approach to the conventional method was noted, separate and solitary use of the RTLS has not been verified. The fact that nearly 40.0% (435/1088) of contact cases and 30.3% (23/76) of secondary contact cases could have been missed without the conventional method is worth noting. Based on the results of the analysis (Table 1) and the Kaplan-Meier curve (Multimedia Appendix 3), we recommend using the RTLS when tracing the contacts of persons with highly contagious diseases, who are likely to wear tags, such as nursing staff, and who share the same space for a long time.

Factors indicative of prolonged exposure to the symptomatic source, such as room sharing and mask-wearing behavior, were not associated with secondary transmission, which was inconsistent with the findings of previous studies [28,29]. On the other hand, being female increased the risk of secondary transmission. This may be because nursing staff members were mostly women at the institution and were involved in activities that had high risks of transmission. Detailed information should be collected to discuss the risk of transmission.

It needs to be noted that contact duration was not statistically different between the 2 groups. This study has advantages in determining the significance of contact duration in the transmission of the disease owing to the implementation of methods capable of quantifying time precisely. The average time spent with confirmed patients was long, which is plausible, considering the interactions taking place between individuals in health care facilities. Maintaining strict precautions, such as frequent hand washing, would be crucial for preventing disease spread when the cumulative time surpasses a certain extent, taking into consideration previous studies that emphasized the role of fomites in disease transmission [30,31]. Furthermore, there were 3 cases of transmission with less than 15 minutes of contact time, which has been designated as a transmission cutoff by the CDC. Considering that the number of participants working in the high-risk department was not significantly different (data not shown), aerosol-producing procedures were not attributable to the finding, even though there was a risk of transmission owing to high-risk behaviors, such as coughing and sneezing. In light of previous reports indicating the failure of containment of the disease with existing guidelines [32], further efforts to elucidate the threshold of the transmission of SARS-CoV-2 are warranted. The RTLS could be used for research purposes to better characterize the transmission rate of a novel disease or variant, thereby guiding institutional and government policies.

### Limitations

This study has some limitations that must be acknowledged. First, the assumption of index cases may not be completely accurate without a genetic analysis [25,29], especially considering the high incidence of COVID-19 cases in the community. Second, owing to the retrospective design of the study, we could not confirm the extent of use of personal protective equipment and the presence of symptoms, especially for the contact cases identified by the RTLS. Third, we could not accurately calculate the positive predictive value of the RTLS contact tracing model because of the lack of verification. Finally, we should take into consideration the cost of installation of the RTLS, which may not be feasible in a resource-limited setting. However, this study is significant in that it investigated the utility of a novel technology in contact tracing in the backdrop of a hospital environment, reflecting the real-world circumstances where disease transmission actually takes place. Our findings underscore the need for further studies investigating the efficiency of the technology with prospectively collected data.

### Conclusions

This study showed that novel technologies, such as the RTLS, are beneficial when used as an adjunctive approach to the conventional method for contact tracing, especially when individuals share rooms with each other and under the influence of highly transmissible diseases. However, the RTLS cannot completely replace the traditional contact tracing method.

## Appendix

Multimedia Appendix 1 Baseline characteristics of participants with and without secondary SARS-CoV-2 transmission.

Multimedia Appendix 2 Comparison of contact tracing methods among secondary transmission cases.

Multimedia Appendix 3 Kaplan-Meier curve for the risk of secondary transmission according to the tracing method based on the study date. The cumulative hazard of secondary transmission is shown for the RTLS method vs the conventional method (*P*=.001). RTLS: real-time locating system.

Multimedia Appendix 4 Subgroup analysis involving contact cases that had follow-up SARS-CoV-2 polymerase chain reaction results within 14 days.

## Abbreviations

aOR: adjusted odds ratio
CDC: Centers for Disease Control and Prevention
PCR: polymerase chain reaction
RFID: radio-frequency identification
RTLS: real-time locating system
RT-PCR: reverse transcription polymerase chain reaction
