# Supplementary material for: The Potential Role of an Adjunctive Real-Time Locating System in Preventing Secondary Transmission of SARS-CoV-2 in a Hospital Environment: Retrospective Case-Control Study
Source: J Med Internet Res. 2022 Oct 18;24(10):e41395. doi: 10.2196/41395 (PMC9580994; doi:10.2196/41395)
Supplement: Multimedia Appendix 1 [file jmir_v24i10e41395_app1.docx]

**Multimedia Appendix 1.** Baseline characteristics of participants with and without secondary SARS-CoV-2 transmission.

|  | All (n=1,088) | Secondary transmission  (N=76) | | Non-  transmission  (N=1,012) | *P* value | | |
| --- | --- | --- | --- | --- | --- | --- | --- |
| **Age (years)** | 41.5±17.5 | 45.4±20.9 | | 41.3±17.1 | .001 | | |
| **Sex (male)** | 275 (25.3) | 14 (18.4) | | 261 (25.8) | .202 | | |
|  |  |  | |  |  | | |
| **Exposure duration (minutes)** | 240 [41-1675.8] | 630 [72.5-1510.5] | | 240 [41-1678] | .123 | | |
| Unknown^a^ | 346 |  | |  |  | | |
|  |  |  | |  |  | | |
| **Personal protective equipment used** | | |  |  | |  |  |
| Mask | 339 (33.1) | 11 (15.7) | | 328 (34.4) | .001 | | |
| Glove | 6 (3.6) | 0 (0.0) | | 6 (3.6) | .737 | | |
| Face shield | 1(0.6) | 0 (0.0) | | 1 (0.6) | .893 | | |
| Unknown^a^ | 64 |  | |  |  | | |
|  |  |  | |  |  | | |
| **Mask wearing consistency^b^** |  |  | |  |  | | |
| At all times | 138 (84.7) | 2 (50.0) | | 136 (85.5) | .051 | | |
| More than 50% | 10 (6.1) | 0 (0.0) | | 10 (6.3) | .605 | | |
| Less than 50% | 15 (9.2) | 2 (50) | | 13 (8.2) | .004 | | |
| Unknown^a^ | 925 |  | |  |  | | |
|  |  |  | |  |  | | |
| **Level of exposure** |  |  | |  |  | | |
| High | 436 (63.5) | 36 (72.0) | | 400(62.9) | .224 | | |
| Intermediate | 198 (28.9) | 14 (28.0) | | 184 (28.9) | 1.000 | | |
| Low | 52 (7.6) | 0 (0.0) | | 52 (8.2) | .026 | | |
| Unknown^a^ | 402 |  | |  |  | | |
|  |  |  | |  |  | | |
| **Type of occupation** |  |  | |  |  | | |
| HCWs | 769 (77.1) | 46 (71.9) | | 723 (77.5) | .301 | | |
| Doctor | 48 (4.8) | 1 (1.6) | | 47 (5.0) |  | | |
| Nurse | 550 (55.2) | 35 (54.7) | | 515 (55.2) |  | | |
| Patient | 228 (22.9) | 18(28.1) | | 210(22.5) |  | | |
| Patient | 171 (17.2) | 14 (21.9) | | 157 (16.8) |  | | |
| Caregiver | 57 (5.7) | 4 (6.3) | | 53 (5.7) |  | | |
| Unknown^a^ | 92 |  | |  |  | | |
|  |  |  | |  |  | | |
| **Type of occupation of index patient** | | |  |  | |  |  |
| HCW | 843 (80.3) | 25 (32.9) | | 182 (18.7) | .003 | | |
| Patient | 207 (19.7) | 51 (67.1) | | 793 (81.3) |  | | |
| Unknown^a^ | 38 |  | |  |  | | |
|  |  |  | |  |  | | |
| **Vaccination status** |  |  | |  |  | | |
| Vaccinated more than once | 741 (83.8) | 13 (25.0) | | 130 (15.6) | .075 | | |
| Days from last vaccination (days)^c^ | 82 [54-82] | 95 [79.75-265.3]) | | 81 [54-170] | .002 | | |
| Unknown^a^ | 192 |  | |  |  | | |
|  |  |  | |  |  | | |
| **Post exposure measure** |  |  | |  |  | | |
| Quarantined | 128 (66.0) | 16 (64.0) | | 112 (66.3) | .823 | | |
| Monitored actively | 34 (17.5) | 0 (0.0) | | 34 (20.1) | .014 | | |
| Monitored passively | 32 (46.5) | 9 (36.0) | | 23 (13.6) | .005 | | |
| Unknown^a^ | 894 |  | |  |  | | |
|  |  |  | |  |  | | |
| **Conventional method** | 509 (46.8) | 27 (35.5) | | 482 (47.6) | .041 | | |
|  |  |  | |  |  | | |
| **Room sharing** | 736 (71.9) | 61 (87.1) | | 675 (70.8) | .003 | | |
| Unknown^a^ | 65 |  | |  |  | | |
|  |  |  | |  |  | | |

Data are expressed as mean ± standard deviation, median [IQR], or number with percentages.

*Abbreviations*: HCW; health care worker, IQR; interquartile range

^a^ Unknown represents number of missing values

^b^ Extent to which each participant conforms to the mask-wearing precaution

^c^ Days passed from the last vaccination
