# Supplementary material for: The Potential Role of an Adjunctive Real-Time Locating System in Preventing Secondary Transmission of SARS-CoV-2 in a Hospital Environment: Retrospective Case-Control Study
Source: J Med Internet Res. 2022 Oct 18;24(10):e41395. doi: 10.2196/41395 (PMC9580994; doi:10.2196/41395)
Supplement: Multimedia Appendix 2 [file jmir_v24i10e41395_app2.docx]

**Multimedia Appendix 2.** Comparison of contact tracing methods among secondary transmission cases.

|  | RTLS exclusive (n=49) | Conventional exclusive (n=23) | Both (n=4) | *P* value |
| --- | --- | --- | --- | --- |
| **Age (years)** | 34.8±13.9 | 67.8±14.5 | 46.2±26.1 | .091^b^ |
| **Sex (male)** | 5 (35.7) | 8 (57.1) | 1 (7.1) | .040 |
|  |  |  |  |  |
| **Exposure duration (minutes)** | 630 [78.25-1527] | 10 [10-15] | 528 [63-983.3] | .136 ^b^ |
|  |  |  |  |  |
| **Level of exposure** |  |  |  |  |
| High | 34 (75.6) | 0 (0.0) | 2 (50.0) | .093 |
| Intermediate | 11 (24.4) | 1 (100.0) | 2 (50.0) | .093 |
| Low | 0 (0.0) | 0 (0.0) | 0 (0.0) | 1.000 |
|  |  |  |  |  |
| **Type of occupation** |  |  |  |  |
| HCWs | 41 (91.1) | 2 (13.3) | 3 (75.0) | <.001 |
| Doctor | 1 (2.2) | 0 (0.0) | 0 (0.0) |  |
| Nurse | 32 (71.1) | 1 (6.7) | 2 (50) |  |
| Patient | 4 (8.9) | 13 (86.7) | 1(25.0) |  |
| Patient | 4 (8.9) | 9 (60) | 1 (25.0) |  |
| Caregiver | 0 (0.0) | 4 (26.7) | 0 (0.0) |  |
|  |  |  |  |  |
| **Type of occupation of index patient** | |  |  |  |
| HCWs | 48 (95.9) | 4(15.4) | 3 (75.0) | <.001 |
| Patient | 2 (4.1) | 22 (84.6) | 1(25.0) |  |
|  |  |  |  |  |
| **Vaccination** |  |  |  |  |
| Number of vaccination doses | 33 (73.3) | 7 (77.8) | 3 (100.0) | .574 |
| Days from last vaccination (days)^c^ | 95 [82-266] | 52 [50.265-1025] | 134 [87.25-179.3] | .902 ^b^ |
|  |  |  |  |  |
| **Post exposure measure** |  |  |  |  |
| Quarantined | 0 (0.0) | 8 (34.8) | 1 (33.3) | .960 |
| Monitored actively | 0 (0.0) | 0 (0.0) | 0 (0.0) | NA |
| Monitored passively | 0 (0.0) | 15 (65.2) | 2 (66.7) | .960 |
|  |  |  |  |  |

Data are expressed as mean ± standard deviation, median [IQR], or number with percentages.

*Abbreviations*: HCW; health care worker, NA; not applicable, IQR: interquartile range

a One-way repeated-measures one-way analysis of variance (ANOVA).

^b^ Days passed from the last vaccination
