# Supplementary figures and images for: The Potential Role of an Adjunctive Real-Time Locating System in Preventing Secondary Transmission of SARS-CoV-2 in a Hospital Environment: Retrospective Case-Control Study
Source: J Med Internet Res. 2022 Oct 18;24(10):e41395. doi: 10.2196/41395 (PMC9580994; doi:10.2196/41395)

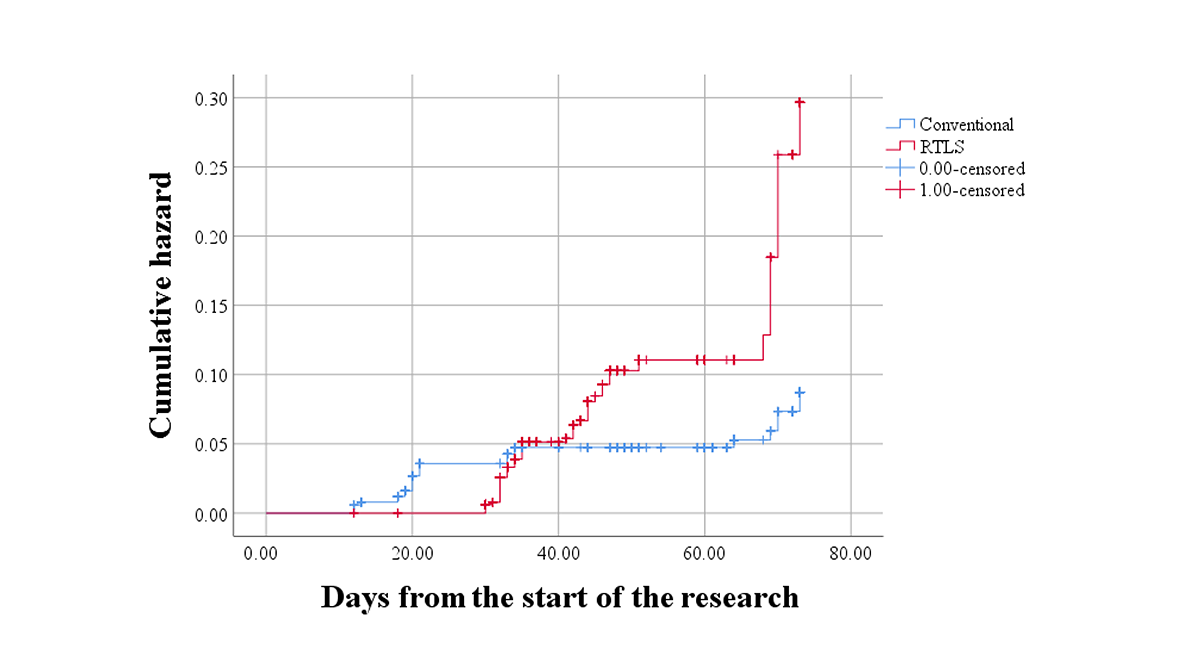

Supplement: Multimedia Appendix 3 [file jmir_v24i10e41395_app3.png]
