# Supplementary material for: The Potential Role of an Adjunctive Real-Time Locating System in Preventing Secondary Transmission of SARS-CoV-2 in a Hospital Environment: Retrospective Case-Control Study
Source: J Med Internet Res. 2022 Oct 18;24(10):e41395. doi: 10.2196/41395 (PMC9580994; doi:10.2196/41395)
Supplement: Multimedia Appendix 4 [file jmir_v24i10e41395_app4.docx]

**Multimedia Appendix 4.** Subgroup analysis involving contact cases that had follow-up SARS-CoV-2 polymerase chain reaction results within 14 days.

|  | univariate | | | multivariable^a^ | | |
| --- | --- | --- | --- | --- | --- | --- |
|  | OR | 95% CI | *P* value | OR^a^ | 95% CI | *P* value |
| Age | 0.98 | 0.94-1.01 | .167 | 1.02 | 0.97-1.07 | .388 |
| Male (ref.Female) | 0.14 | 0.01-0.69 | .059 | 0.19 | 0.01-1.00 | .115 |
| Days from last vaccination (days)^b^ | 1.05 | 1.02-1.08 | .001 | 1.04 | 1.01-1.07 | .016 |
| Room sharing | 1.96 | 0.84-5.35 | .148 | 1.52 | 0.25-7.72 | .622 |
| Mask wearing | 0.44 | 0.18-0.97 | .052 | 1.31 | 0.23-5.56 | .736 |
| RTLS (ref. conventional) | 15.09 | 5.27-63.65 | <.001 | 14.20 | 4.44-65.47 | <.001 |

Logistic regression was used to calculate the risk of secondary transmission. Variables with clinical significance and statistical significance in univariate analysis were included in the model. Abbreviations: OR; odds ratio, CI; confidence interval, RTLS; real time locating system, ref.; reference

^a^ Multivariable analysis adjusted for all the variables involved in univariate model.

^b^Days passed from the last vaccination
